# Cowden Syndrome in Childhood: Gastrointestinal Involvement in a Multisystem Genetic Disorder—A Case Report

**DOI:** 10.3390/reports9010021

**Published:** 2026-01-09

**Authors:** Maria Rogalidou, Nikolaos Katzilakis, Kalliopi Stefanaki, Konstantina Dimakou, Dafni Margoni, Iordanis Pelagiadis, Alexandra Papadopoulou, Eftichia Stiakaki

**Affiliations:** 1 Division of Gastroenterology & Hepatology, First Department of Paediatrics, National and Kapodistrian, University of Athens ‘Aghia Sophia’ Children’s Hospital, 11527 Athens, Greece; 2 Department of Pediatric Hematology-Oncology, University Hospital of Heraklion, 71110 Crete, Greece; 3 Pathology Department, Agia Sofia Children’s Hospital, 11527 Athens, Greece; 4 Gastroenterology Department, Agia Sofia Children’s Hospital, 11527 Athens, Greece; 5 First Department of Paediatrics, National and Kapodistrian, University of Athens ‘Aghia Sophia’ Children’s Hospital, 11527 Athens, Greece

**Keywords:** Cowden syndrome, cancer predisposition, multiple hamartoma syndrome, gastrointestinal polyposis, PTEN gene mutation

## Abstract

**Background and Clinical significance:** Cowden syndrome is an autosomal dominant disorder caused by germline loss-of-function mutations in the PTEN tumor suppressor gene. It is characterized by multiple hamartomas and an increased lifetime risk of malignancies affecting the breast, thyroid, endometrium, and gastrointestinal (GI) tract. Pediatric presentations may include macrocephaly, scrotal tongue, and intellectual disability. Gastrointestinal involvement is frequent, with juvenile-like hamartomatous polyps occurring in at least half of patients and distributed throughout the GI tract, posing a risk for malignant transformation. Early diagnosis and surveillance are crucial for improving patient outcomes. **Case Presentation:** We report a case of a 10-year-old Caucasian female with Cowden syndrome, with a history of a malignant germ cell tumor of the ovary consisting of a yolk sac tumor and low-grade immature teratoma diagnosed at age six, and thyroidectomy at age nine. The patient has mild intellectual disability. Routine radiological surveillance revealed a right colon intraluminal mass, prompting referral for pediatric gastroenterology evaluation. Endoscopy identified multiple polyps throughout the colon, stomach, and small intestine. Polypectomy of larger lesions was performed, and histopathology confirmed juvenile-like hamartomatous polyps without dysplasia or malignancy. This case highlights the necessity of comprehensive gastrointestinal evaluation in pediatric Cowden syndrome patients. Endoscopic surveillance is essential for early detection and management of polyps. **Conclusions:** Given the multisystem involvement and elevated cancer risk associated with PTEN mutations, a multidisciplinary approach that includes genetic counseling, dermatologic evaluation, and ongoing oncologic monitoring is recommended. Increased awareness of gastrointestinal manifestations enables timely intervention and may reduce morbidity and mortality in this high-risk population.

## 1. Introduction and Clinical Significance

Cowden syndrome (CS) is considered the prototypical clinical phenotype of the broader PTEN hamartoma tumor syndrome (PHTS) spectrum which also includes Bannayan–Riley–Ruvalcaba Syndrome (BRRS), PTEN-related Proteus Syndrome (PS), and Proteus-like Syndrome [1]. It is a rare, multisystem genetic disorder (autosomal dominant disease) with an estimated prevalence of 1 in 200,000–250,000 individuals, with de novo pathogenic mutations occurring in approximately 10–30% of cases. It is caused by germline mutations in the PTEN tumor suppressor gene located on chromosome 10q23 [1]. Tan et al. [2] examined genotype–phenotype correlations in children and adolescents younger than 18 years to establish pediatric clinical criteria for PTEN testing. Their findings indicated that macrocephaly—defined as an occipitofrontal circumference exceeding two standard deviations above the population mean (97.5th percentile)—was an essential diagnostic feature, as it was present in 100% of patients at the time of diagnosis [2]. Neurological manifestations, including autism spectrum disorder and developmental delay, along with dermatological findings such as lipomas and oral papillomas, were identified as highly prevalent secondary characteristics; involvement of one or both of these systems was observed in all individuals with germline PTEN mutations [2]. Additional clinical features that were less frequently noted at initial pediatric presentation included vascular abnormalities (such as arteriovenous malformations), gastrointestinal polyps, thyroid enlargement, and early-onset malignancies, including thyroid and germ cell cancers, which are likely to be less common in early childhood.

The PTEN (phosphatase and tensin homolog) gene is a well-established tumor suppressor, located on chromosome 10q23.3. It encodes a dual-specificity phosphatase that plays a crucial role in regulating cellular processes such as growth, proliferation, survival, and migration, primarily through negative regulation of the PI3K/AKT/mTOR signaling pathway. Germline or somatic mutations in PTEN result in loss of function of the PTEN protein. This dysfunction leads to accumulation of phosphatidylinositol (3,4,5)-trisphosphate (PIP3), causing constitutive activation of downstream signaling effectors such as AKT and mTOR. The resulting hyperactivation promotes uncontrolled cellular proliferation, decreased apoptosis, and enhanced cell survival, contributing to tumorigenesis and the development of hamartomatous lesions [3].

CS is characterized by a wide range of clinical manifestations affecting the dermatologic, neurologic, endocrine, and gastrointestinal systems (Table 1). In children, early signs of the disease may include progressive macrocephaly, facial dysmorphism, and mild to moderate developmental delays, while mucocutaneous features such as trichilemmomas, scrotal tongue, and palmar or plantar hyperkeratotic pits typically appear later in childhood. PHTS should be therefore suspected in children presenting with macrocephaly accompanied by one or more of the following features: autism spectrum disorder or developmental delay; dermatologic findings such as lipomas, trichilemmomas, oral papillomas, or penile freckling; vascular anomalies, including arteriovenous malformations or hemangiomas; gastrointestinal polyps; pediatric-onset thyroid cancer or germ cell tumors.

Due to its heterogeneous presentation, formal clinical diagnostic criteria [1,2,4,5] have been developed and are regularly updated to reflect ongoing advances in the understanding the syndrome. These criteria are particularly important when a pathogenic PTEN mutation has not been confirmed. The current diagnostic criteria [2], in the absence of molecular confirmation, are as follows. Clinical diagnosis can be made if: A. No PTEN mutation is found, and one of the following applies: a.—Three or more major criteria, with at least one being macrocephaly, Lhermitte-Duclos disease, or GI hamartomas; b.—Two major criteria and three or more minor criteria. B. In the presence of a PTEN mutation, phenotypic variation is broad. Any one or more major features, or multiple minor features, confirm the diagnosis.

## 2. Case Presentation

We present a 10-year-old girl with a complex medical background and a family history including tuberous sclerosis in her mother and older brother (the latter also with epilepsy and intellectual disability), colorectal cancer in her maternal grandfather at age 47, and thyroid cancer in her father. She was born prematurely at a gestational age of 31 weeks, with a birth weight of 1900 g, a length of 44 cm, and a head circumference of 33.5 cm, following in vitro fertilization using donor eggs. In early infancy, she was initially evaluated for macrocephaly (head circumference at 6 months: 46 cm; at 20 months: 56 cm, >97th percentile for age) and mild intellectual disability. She began speech therapy and occupational therapy at the age of two years. Her brain MRI was normal, and no initial unifying diagnosis was made.

**Figure 1:**
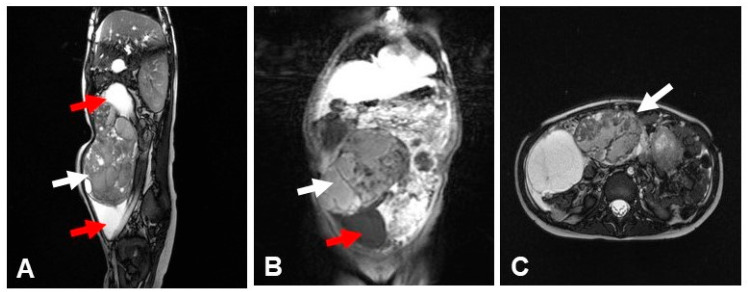
Imaging of the abdomen with the abdominal mass. (**A**): T2 sagittal: large lesion measuring 19 cm × 15.5 cm × 6.8 cm, with cystic components and internal septations. (white arrow). At the inferior and superior margins, large cysts are observed, measuring 6.3 cm and 7.3 cm in diameter (red arrows); (**B**): T1 coronal: A large mass, consistent with a teratoma of the left ovary (red arrow). (**C**): A large mass with cystic components and internal septations (arrow) located posterior to the abdominal walls.

**Figure 2:**
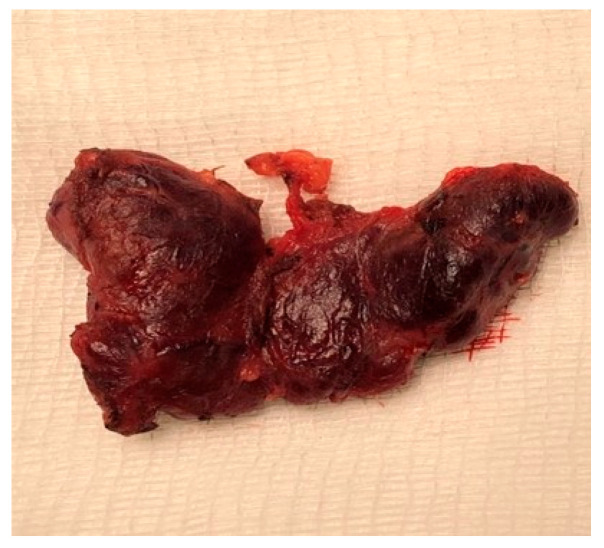
Thyroid gland after thyroidectomy.

At six years, she presented with a large intra-abdominal mass (Figure 1); imaging and biopsy revealed a malignant germ cell tumor of the right ovary composed of a yolk sac tumor with omental metastases, along with a synchronous low-grade immature teratoma in the left ovary, and laboratory tests showed AFP >20,000 ng/mL with normal β-hCG. She was treated with surgery, chemotherapy, and radiotherapy according to the International SIOP non-CNS GCT protocol and has remained disease-free for seven years under pediatric oncology surveillance. At nine years, she developed hoarseness and palpable cervical lymph nodes; thyroid ultrasound raised suspicion of malignancy, and FNA of nodules N1 and N2 demonstrated nodular thyroid hyperplasia with focal atypia (Bethesda III, AUS/FLUS). Therefore, a total thyroidectomy (Figure 2) was performed which revealed nodular hyperplasia with hyperplastic and colloid nodules and no evidence of carcinoma. Given the combination of macrocephaly, thyroid findings, and prior germ cell tumor, clinical exome sequence analysis was performed and identified a heterozygous pathogenic PTEN variant (c.1003C>T; p.Arg335Ter), confirming Cowden syndrome/PTEN hamartoma tumor syndrome. Further genetic testing of the child or father was not performed due to financial constraints.

**Figure 3:**
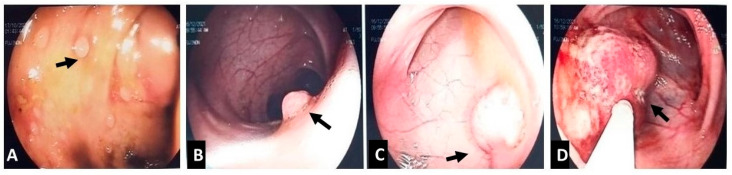
Multiple polyps throughout the colon, and polypectomy (arrows). (**A**) multiple small polyps. (**B**) sessile polyp. (**C**) pedunculated polyp. (**D**) polypectomy.

**Figure 4:**
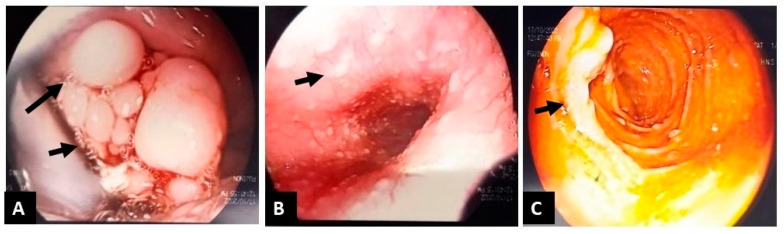
(**A**): Multiple polyps the basis of tongue (arrows); (**B**): features of glycogenous acanthosis of the esophagus (arrow); (**C**): Polyps of duodenum, particularly near the ampulla of Vater (arrow).

**Figure 5:**
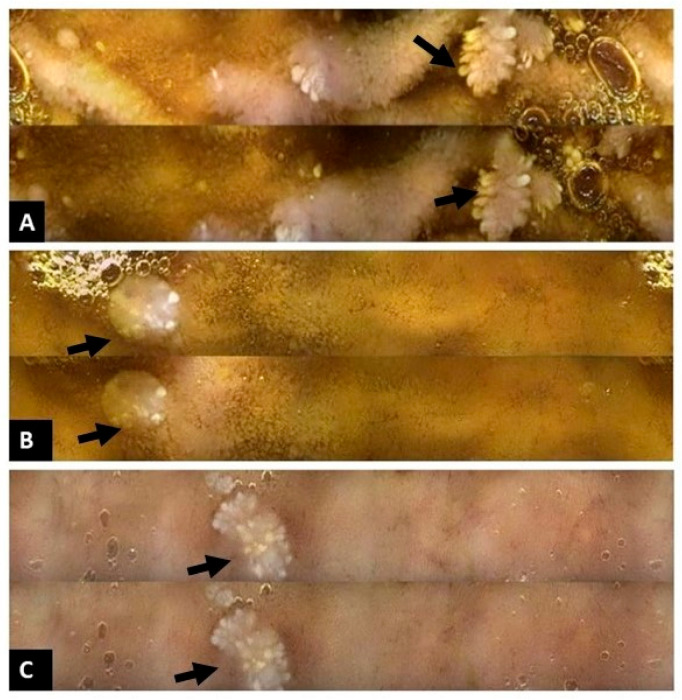
Capsule endoscopy images: Polyps in the small intestine; (**A**): small polyps in jejunum (arrows); (**B**): small polyps in Ileum (arrows); (**C**): small polyps in terminal ileum (arrows).

At age ten, routine follow-up imaging detected a right-colon intraluminal mass, and colonoscopy revealed multiple colorectal polyps, approximately 15 of which were removed (Figure 3); histology showed juvenile-like hamartomatous polyps without dysplasia. Six months later, upper and repeat lower endoscopy, together with capsule endoscopy, demonstrated multiple polyps at the base of the tongue (Figure 4A), early glycogenic acanthosis of the esophagus (Figure 4B), numerous gastric and duodenal polyps, particularly near the ampulla of Vater (Figure 4C), diffuse colonic polyposis, and small intestinal polyps (Figure 5).

She is currently enrolled in a structured gastrointestinal surveillance program with annual endoscopic evaluations initially, then every two to three years or sooner if symptoms arise, and extended genetic counseling and germline testing have been recommended for at-risk family members.

A consent form from parents was obtained for anonymized publication of the patient’s history and images of endoscopic, histological and radiological findings.

## 3. Discussion

This case report of a child with CS highlights the important role of pediatric gastroenterologists in the early identification, diagnosis, and long-term management of this syndrome. Our patient was found to have a heterozygous pathogenic PTEN variant (c.1003C>T; p.Arg335Ter), confirming the diagnosis of Cowden syndrome. According to the American College of Medical Genetics and Genomics (ACMG) guidelines, this variant is classified as pathogenic based on multiple criteria, including PVS1 (null variant in a gene where loss of function is a known disease mechanism), PS3 (well-established functional studies), PM2 (absence from population databases), PP3 (computational evidence supporting a deleterious effect), and PP5.

Pediatric Cowden syndrome shows significant clinical heterogeneity across neurological, endocrine, gastrointestinal, and dermatological domains. Clinical features display clear age-dependent patterns: mucocutaneous lesions are typically absent at diagnosis [6] and increase with age [7], while macrocephaly and developmental concerns often serve as early diagnostic indicators. This pronounced phenotypic variability, even among patients with identical mutations [8], suggests that additional genetic modifiers or environmental factors influence clinical expression.

In a recent study of 11 pediatric patients with PHTS [9], the most common diagnoses before genetic testing were macrocephaly (11/11) and developmental delay (5/11). Similarly, our patient presented with macrocephaly and mild developmental delay, which, although subtle now, was more apparent earlier and has improved with supportive therapies. Macrocephaly is nearly universal (85%) [10], with an average head circumference of +5.7 SD [6]. In contrast, neurodevelopmental features vary considerably, ranging from normal development [11] to severe intellectual disability with autism [12], with autism spectrum disorder affecting 27–50% of patients [10].

Thyroid involvement affects 52% of screened patients [10], although malignancy mainly occurs after age 10 and consistently exhibits low invasive behavior [10].

Tongue polyps, especially at the base of the tongue, have been reported in children with Cowden Syndrome, although they are more frequently described in adults. These mucosal hamartomas are part of the mucocutaneous spectrum of CS and may serve as an early clinical clue [13]. In our patient, multiple asymptomatic polyps between the pharynx and the base of the tongue were observed during upper GI endoscopy, reinforcing the diagnosis of CS.

Gastrointestinal manifestations (Table 2) range from asymptomatic polyps to life-threatening hemorrhage requiring colectomy, with early progression to dysplasia by ages 2 to 4 [14].

Glycogenic acanthosis of the esophagus, a benign epithelial proliferation, is commonly reported in adult CS patients [15], but appears less frequent in children. In a cohort study of 80 pediatric patients with confirmed PTEN mutations, lymphoid hyperplasia and eosinophilic gastrointestinal disorders (EGID) were observed in 14% and 6% of cases, respectively. Another common histological finding was nonspecific mucosal inflammation, including esophagitis, gastritis, and duodenitis.

Furthermore, while surveillance in adults typically focuses on colorectal cancer risk, emerging data suggest that gastrointestinal polyposis may be one of the earliest clinical manifestations in children with CS [16,17].

Atypical manifestations include focal cortical dysplasia with epilepsy [18], toxic thyroid adenoma causing hyperthyroidism [19], and early-onset protein-losing enteropathy at 3 to 6 months [14].

Children with CS or PHTS have a lifetime risk of developing both benign and malignant tumors. The malignancy spectrum extends beyond thyroid cancer to include rare pediatric cases: ovarian dysgerminoma at age 7 [20], renal cell carcinoma at age 11 [21], and three germ cell tumors [22].

Early surveillance and routine monitoring are essential, even though most cancers associated with these conditions typically appear in adolescence or adulthood. Gastrointestinal and other systemic features may present during childhood, providing early diagnostic clues and enabling timely intervention. A recent review highlights the importance of early gastrointestinal surveillance in children with confirmed PTEN mutations [10]. However, recommendations from different organizations and working groups for surveillance of children with PTEN mutations differ slightly. GENTURIS guidelines [23] recommend a colonoscopy every five years starting at age 35, or earlier if symptoms are present or if a close relative had colorectal cancer before age 40. The American Association for Cancer Researchers [24] recommends annual colonoscopy based on symptoms. The International PHTS Cancer and Overgrowth Guidelines Working Group, consisting of six international specialists in the diagnosis and management of PHTS [25], advises that patients with PHTS who exhibit a high colorectal polyp burden—defined as five or more tubular adenomas—polyps measuring at least 1 cm, or lesions demonstrating high-grade dysplasia should receive surveillance colonoscopy every one to three years, according to the discretion of their treating gastroenterologist.

## 4. Conclusions

This case highlights the importance of recognizing Cowden syndrome as a multisystem disorder that can present early in childhood with gastrointestinal polyposis, developmental delay, and malignancies. Early genetic diagnosis of PTEN mutations enables prompt initiation of surveillance strategies, which is crucial for reducing cancer risk and managing complications. Multidisciplinary care and increased clinical awareness are essential for improving long-term outcomes in pediatric patients with Cowden syndrome or other PTEN-related disorders.

## Figures and Tables

**Table 1:** Clinical Diagnostic Criteria (If No Confirmed PTEN Mutation).

Major Criteria	Minor Criteria
Breast cancer	Autism spectrum disorder
Endometrial cancer (epithelial)	Intellectual disability/developmental delay
Thyroid cancer (papillary or follicular)	Lipomas
Multiple GI hamartomas or ganglioneuromas (≥3)	Fibrocystic breast disease
Macrocephaly (≥97th percentile)	Uterine fibroids
Lhermitte-Duclos disease (cerebellar dysplastic gangliocytoma)	Vascular anomalies (AVMs, hemangiomas)
Mucocutaneous lesions: Trichilemmomas (facial) Acral keratoses Papillomatous papules (esp. oral mucosa) Mucosal lesions (oral papillomas)	Esophageal glycogenic acanthosis (≥3)
	Colon adenomas
	Renal cell carcinoma
	Testicular lipomatosis

**Table 2:** Gastrointestinal Manifestations in children with Cowden Syndrome.

Feature	Description
Prevalence	~28–50% of children with PTEN mutations have GI manifestations
Polyp burden	Variable: from few scattered to extensive polyposis across the GI tract
Polyp location	Colon, rectum, stomach, duodenum, esophagus, small intestine
Polyp types	Mixed histology: ▪ Juvenile-like hamartomas (most common) ▪ Inflammatory polyps ▪ Hyperplastic polyps ▪ Ganglioneuromas ▪ Adenomatous polyps (occasionally)
Other less common	Glycogenic acanthosis of esophagus
Other findings	Nonspecific inflammation: esophagitis, gastritis, duodenitis Lymphoid hyperplasia EGIDs (Eosinophilic Gastrointestinal disorders)
Symptoms	Often asymptomatic; when present: ▪ Constipation ▪ Feeding difficulties, food aversion, aspiration ▪ Gastroesophageal Reflux disease ▪ Rectal bleeding ▪ Abdominal pain ▪ Iron-deficiency anemia ▪ Diarrhea ▪ Failure to thrive

## Data Availability

The original contributions presented in this study are included in the article. Further inquiries can be directed to the corresponding author.

## Abbreviations

The following abbreviations are used in this manuscript:

PTEN	Phosphatase and TENsin homolog
CS	Cowden Syndrome
PHTS	PTEN Hamartoma Tumor Syndrome
GI	Gastrointestinal
MRI	Magnetic Resonance Imaging
BRRS	Bannayan–Riley–Ruvalcaba Syndrome
PTEN PS	PTEN-related Proteus Syndrome
FNA	Fine-Needle Aspiration
AUS	Atypia of Undetermined Significance
FLUS	Follicular Lesion of Undetermined Significance
